# Genotyping of *Chlamydophila psittaci *using a new DNA microarray assay based on sequence analysis of *omp*A genes

**DOI:** 10.1186/1471-2180-8-63

**Published:** 2008-04-17

**Authors:** Konrad Sachse, Karine Laroucau, Helmut Hotzel, Evelyn Schubert, Ralf Ehricht, Peter Slickers

**Affiliations:** 1 Friedrich-Loeffler-Institut (Federal Research Institute for Animal Health), Insitute of Molecular Pathogenesis, Jena, Germany; 2 AFSSA, Unité Zoonoses Bactériennes, Maisons-Alfort, France; 3 Friedrich-Loeffler-Institut (Federal Research Institute for Animal Health), Insitute of Bacterial Infections and Zoonoses, Jena, Germany; 4 Clondiag Chip Technologies, Jena, Germany

## Abstract

**Background:**

The currently used genotyping system for the avian zoonotic pathogen *Chlamydophila (C.) psittaci *has evolved from serology and is based on *omp*A sequence variations. It includes seven avian and two non-avian genotypes. Restriction enzyme cleavage of the amplified *omp*A gene and, less frequently, *omp*A sequencing are being used for examination, but, beside methodological limitations, an increasing number of recently tested strains could not be assigned to any established genotype.

**Results:**

Comprehensive analysis of all available *omp*A gene sequences has revealed a remarkable genetic diversity within the species *C. psittaci*, which is only partially covered by the present genotyping scheme. We suggest adjustments and extensions to the present scheme, which include the introduction of subgroups to the more heterogeneous genotypes A, E/B and D, as well as six provisional genotypes representing so far untypable strains. The findings of sequence analysis have been incorporated in the design of a new DNA microarray. The ArrayTube™ microarray-based *omp*A genotyping assay has been shown to discriminate among established genotypes and identify so far untyped strains. Its high specificity, which allows detection of single-nucleotide polymorphisms, is due to the parallel approach consisting in the use of 35 hybridization probes derived from variable domains 2 and 4 of the *omp*A gene.

**Conclusion:**

The traditional genotyping system does not adequately reflect the extent of intra-species heterogeneity in *omp*A sequences of *C. psittaci*. The newly developed DNA microarray-based assay represents a promising diagnostic tool for tracing epidemiological chains, exploring the dissemination of genotypes and identifying non-typical representatives of *C. psittaci*.

## Background

The obligate intracellular bacterium *Chlamydophila (C.) psittaci*, the causative agent of psittacosis in birds and humans, is a well-established pathogen responsible for regular outbreaks of disease in psittacine birds and domestic poultry [1], as well as cases of atypical pneumonia in exposed persons [2,3].

The current definition of the species *C. psittaci *includes the former avian serovars of *Chlamydia psittaci*, but even under the recently revised taxonomic classification of the family *Chlamydiaceae *[4] it remains a heterogeneous taxon in terms of host range and virulence. To facilitate epidemiological studies, strains of the agent were subdivided into serovars A, B, C, D, E, and F on the basis of their immune reaction with a panel of monoclonal antibodies (MAbs) recognising specific epitopes of the major outer membrane protein (MOMP). Each serovar was assumed to exhibit more or less stringent host specificity [5-7]. Later on, Sayada et al. [8] suggested restriction fragment length polymorphism (RFLP), i.e. PCR amplification of the *omp*A gene with subsequent restriction enzyme analysis, for differentiation among *C. psittaci *isolates. Vanrompay et al. [9] were able to demonstrate by comparison of serotyping and PCR-RFLP that the serovars had genetic equivalents in the corresponding genotypes, which were defined by their restriction enzyme cleavage pattern. Thus, nine different genotypes have been generally accepted to date, seven of which are thought to predominantly occur in a particular order or class of *Aves *and two in non-avian hosts, i.e. genotype A in psittacine birds, B in pigeons, C in ducks and geese, D in turkeys, E in pigeons, ducks and others, F in parakeets, WC in cattle, and M56 in rodents. In addition, Geens et al. [10] suggested the introduction of genotype E/B to represent a group of isolates from ducks. Most of the avian genotypes have also been identified sporadically in isolates from cases of zoonotic transmission to humans.

While serotyping can be conducted with cultured strains only, PCR-RFLP was also used with DNA extracted from clinical samples [9]. However, there are obvious limitations as substantial amounts of a PCR amplicon are needed to produce distinctive and reproducible RFLP patterns on ethidium bromide-stained agarose gels. Related genotypes tend to have quite similar patterns, which may be difficult to distinguish, and typing results based on different enzyme patterns (e.g. *Alu*I vs. *Mbo*II) may be contradictory. Genetically aberrant strains cannot be genotyped using the above-mentioned PCR-RFLP procedure. Sequencing of the *omp*A gene and alignment with type strain sequences can also be used to identify the genotype of *C. psittaci *strains [10,11], since genotype-specific sites are located in the gene's variable domains (VD) VD2 and VD4. However, all these approaches have been used in a rather pragmatic manner, i.e. with the aim of working out distinctive features of isolates for epidemiological purposes, while disregarding the scope of natural sequence variability and avoiding a molecular definition of individual genotypes at the nucleotide level.

In this situation, DNA microarray technology can be expected to provide added value because of its highly parallel approach, which implies the potential to exploit minor sequence differences at multiple sites for discrimination among samples [12]. Using the ArrayTube™(AT) platform, we recently demonstrated that the performance parameters of an AT microarray assay for species identification of chlamydiae were comparable to real-time PCR in terms of sensitivity and superior in specificity [13]. This prompted us to take further advantage of the AT technology's high discriminatory capacity [14,12], rapidity and relatively low cost by exploring its suitability for *omp*A genotyping analysis. However, we immediately realized that a comprehensive analysis of currently known *omp*A sequences was necessary before considering the definition of specific probes for each genotype.

In the present study, we report the results of an extensive investigation on sequence similarity among all available *omp*A sequences from species of the genus *Chlamydophila *and describe the development of a DNA microarray-based assay for *omp*A genotyping of *C. psittaci*.

## Results

### Analysis of *ompA *sequences of *Chlamydophila *strains

The NCBI database was searched for *ompA *sequences by repeatedly running BLAST queries with sequences from already known entries. In the course of this process it became evident that some *C. psittaci *sequences were more similar to *omp*A of other *Chlamydophila *spp. than to any genotype of *C. psittaci*, which is in line with earlier publications [15-17]. Therefore, we extended our analysis to include all sequences from the genus *Chlamydophila*.

**Figure 1:**
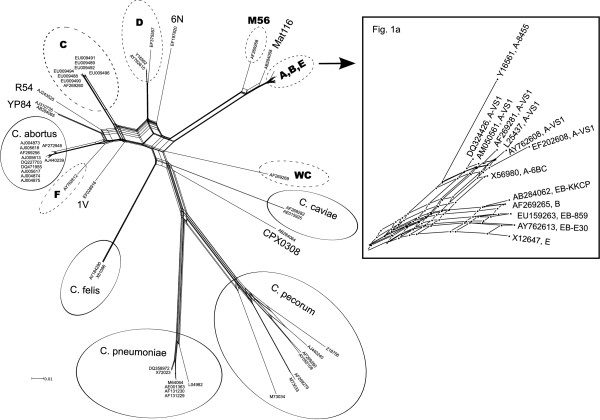
**Split network graph constructed from a global alignment of 63 *ompA *sequences retrieved from GenBank. **Accession numbers are shown for each sequence represented. The length of connecting lines between two items is equivalent to their genetic distance. The scale bar denotes 1 substitution per 100 nucleotides. Clades representing an established genotype of *C. psittaci *are encircled by a dashed line and designated accordingly in bold print. Provisional genotypes are designated as suggested in Table 1. Clades representing other *Chlamydophila *spp. are encircled by a solid line and labeled with the species name. Basic data of the strains represented by accession numbers can be found in Additional file 3. Fig. 1a Detail showing the ABE cluster. Subgroups of genotypes A and E/B are indicated at the respective GenBank accession number.

Comparison of all available GenBank entries revealed 63 unique sequences of *omp*A genes. The alignment of these sequences was the basis for calculation of a sequence similarity matrix (see Additional file 1) and construction of a split network graph (Fig. 1). These graphs are useful tools for presentation of sequence similarity-based relatedness, but are not designed to characterize phylogenetic relationships. We minimized distorting effects due to alignment of differently sized sequences by excluding the shortest items and selecting a 992-nt sequence window which contains all variable domains. The most striking feature of the split network graph is the great diversity among *omp*A variants of *C. psittaci*, which clearly exceeds variations among other species of the genus. While *C. pneumoniae, C. abortus, C. pecorum, C. felis*, and *C. caviae *each are located on a single branch, several genotypes of *C. psittaci *form their own separate branches. At least 12 distinct clades belonging to this species can be identified, of which only 5 can be directly attributed to currently accepted genotypes, i.e. C, D, F, M56, and WC. Another remarkable feature is the ABE cluster, i.e. a grouping harboring the closely related genotypes A, B, E, and E/B. Similarity of the underlying *omp*A sequences is above 98% within the cluster and higher than 99.4% within the clade of genotypes B, E and E/B. To visualize these relationships, a magnified scale was used for presentation in Fig. 1a.

The M56 and Mat116 sequences represent less closely related side branches of the ABE stembranch. Strains of genotypes C, D and F are all located clearly separated from each other, and a number of strains not assigned to one of the accepted genotypes (strains 1V, YP84, R54, 6N, and CPX0308) are placed fairly isolated, forming their own branches at considerable genetic distances from the established genotypes. Genotype F (represented by strain VS225), as well as strains YP84 and R54, were found to be side branches of the *C. abortus *branch.

As a result of this sequence analysis, we identified 20 individual type strains, each of which represents a unique *omp*A sequence. The findings are summarized in Table 1. In this classification, genotypes A, D and E/B can be further divided into subgroups, and the six strains at the bottom of the table represent untyped *C. psittaci *strains.

**Table 1:** Identification of *Chlamydophila psittaci *genotypes and subgroups based on analysis of published *omp*A sequences

**Genotype-Subgroup**	**Type strain**	**GenBank acc. no.**
A-VS1	VS1, MN Zhang	AF269281.1
A-6BC	6BC	X56980.1
A-8455	84–55	Y16561.1
B	CP3	AF269265.1
C	GR9, avian type C	L25436.1
D-NJ1	NJ1	AF269266.1
D-9N	9N	EF375557.1
E	CPMN, EAE A22/M	X12647.1
EB-E30	WS/RT/E30	AY762613.1
EB-859	06-859/1	EU159263.1
EB-KKCP	KKCP-1	AB284062.1
F	VS225	AF269259.1
M56	M56	AF269268.1
WC	WC	AF269269.1
1V*	1V	EF028916.1
6N*	6N	EF197820.1
Mat116*	Mat116	AB284058.1
R54*	R54	AJ243525.1
YP84*	Daruma-1981	AB284065.1
CPX0308*	CPX0308	AB284064.1

* provisional genotypes

All in all, the present sequence analysis has shown that the extent of intra-species variation goes beyond the area covered by currently accepted genotypes.

### Selection of hybridization probes

Using the global alignment of *omp*A sequences (see Additional file 2), we selected a panel of 35 oligonucleotide probes for identification of *C. psittaci *genotypes, which are shown in Table 2. The probe binding sites are located in VD2 and VD4 of the *omp*A gene. A compilation showing the number of mismatches between targets of genotypes and hybridization probes is given in Table 3. These data can be used for two purposes: a) to identify the genotype of the target DNA, and b) to predict signal intensities because perfect matches between probe and target will produce the strongest signals.

**Table 2:** Sequence characteristics of the hybridization probes on the AT genotyping microarray

**Probe**	Sequence*	Binding site*	**Length**	**GC/%**	**Tm/°C**
VD2-01	GGAATTGCTGGAAATAGCGAAAGTAATGC	AF269269.1 [466:494]	29	41	60.9
VD2-02	GGTTTTCAGCTGCAAGCTCAATCTC	M73035.1 [554:578]	25	48	60.3
VD2-03	GGTTTTCAGCTACCAACTCAACCTCT	AF269265.1 [488:513]	26	46	60.3
VD2-04	GGTTTTCAGCTACCAGCTCAACCT	AY327465.1 [488:511]	24	50	60.3
VD2-11	AGGAAACACCTTAACAAATGACCGACT	AJ310735.1 [483:509]	27	41	60.2
VD2-12	GCTACCAACTCAACCTCTACCGATCT	AF269265.1 [496:521]	26	50	61.0
VD2-13	GAGCCTCTTTATCAGAGCAACTTCCA	AJ243525.1 [324:349]	26	46	60.0
VD2-14	AGCTCCTTAACAAATGACCAACTTCCC	AF269260.1 [478:504]	27	44	60.7
VD2-15	CCAGCTCAACCTCTACCGAGCT	AY327465.1 [500:521]	22	59	61.0
VD2-21	CCGTAGCAGCTGATCAACTTCCA	AF269259.1 [482:504]	23	52	60.1
VD2-22	GCAGTTAGTACCGATCTTCCAAAGCA	AF269268.1 [505:530]	26	46	60.5
VD2-23	TCAATAATCAACTTCCAAACGTAGCCATCA	AF269266.1 [494:523]	30	37	60.6
VD2-24	CTCTACCGAGCTTCCAATGCAACT	AY327465.1 [510:533]	24	50	60.2
VD2-25	CTCTACCGATCTTCCAACGCAACT	AF269281.1 [738:761]	24	50	60.0
VD2-31	CGATCTTCCAACGCAACTTCCTAAC	AF269281.1 [744:768]	25	48	59.7
VD2-32	CGATCTTCCAATGCAACTTCCTAACG	M73035.1 [582:607]	26	46	59.9
VD2-33	CGATCTTCCAAAGCAACTTCCTAACG	AF269268.1 [516:541]	26	46	59.8
VD2-34	CGAGCTTCCAATGCAACTTCCTAAC	AY327465.1 [516:540]	25	48	59.9
VD4-01	TGGCTACTGCTGTTTTAGACGCA	AF269269.1 [923:945]	23	48	59.9
VD4-02	TGGCCTCTGCTGTTATGAACTTGAC	AF269260.1 [914:938]	25	48	60.5
VD4-03	AGCCGCTGCTGTTTTGAACTTGA	AF269259.1 [915:937]	23	48	61.2
VD4-11	CCCAAGCCTTATAGGATCAACCACTG	M73035.1 [1047:1072]	26	50	60.3
VD4-12	CCAAGCCTTCTAGGATCAACCACTG	AF269265.1 [982:1006]	25	52	60.3
VD4-13	CCAAGCCTTGTAGGATCAACCACTG	M73035.1 [1048:1072]	25	52	60.8
VD4-21	CCTTATAGGATCAACCACTGCTTTGCC	M73035.1 [1053:1079]	27	48	61.1
VD4-22	CCTTCTAGGATCAACCACTACTTTGCC	AF269262.1 [927:953]	27	48	60.4
VD4-23	CCTTTTAGGGGAAGCCACAAATTTAGACT	AJ243525.1 [799:827]	29	41	60.7
VD4-24	CCTTCTAGGATCAACCACTGCTTTGC	AF269265.1 [987:1012]	26	50	61.2
VD4-25	AGGGCAAGCTACAAATTTAGATACTAGCA	AJ310735.1 [969:997]	29	38	59.7
VD4-31	ACTACTTTGCCCAATAATGGTGGTAAGG	AF269262.1 [943:970]	28	43	60.4
VD4-32	CTGCTTTGCCCAATAATAATAGTGGTAAGG	Y16561.1 [1004:1033]	30	40	59.8
VD4-33	TGCTTTGCCCAATAATAGTGGTAAGGA	M73035.1 [1071:1097]	27	41	59.8
VD4-34	AGCTTTAGATGCTAGCAACAAATTCTGC	AF269259.1 [972:999]	28	39	60.1
VD4-35	GCTTTGCCCAATAATGCTGGTAAGG	AY327465.1 [1006:1030]	25	48	60.2
VD4-36	TGTCGACGGTACCAATACTTACTCTGA	AF269266.1 [981:1007]	27	44	60.3

mean			25	47	60.4

*The exact binding position is referenced by the uniform sequence address (USA). This is the standard sequence notation scheme of the emboss software suite .

**Table 3:** Matching parameters of genotypes and subgroups with probes used on the AT microarray (numbers represent mismatches)

**Probe category**	ABE:VD2-a			ABE:VD2-b		ABE:VD2-c		ABE:VD2-d				ABE:VD4-b			ABE:VD4-c			ABE:VD4-d				CDFRY:VD2-b			CDFRY:VD2c		CDFRY:VD4-c		CDRFY:VD4-d		CF:VD4-a		M56:VD2-c	WC:VD2-a	WC:VD4-a
**Probe**	VD2-02	VD2-03	VD2-04	VD2-12	VD2-15	VD2-24	VD2-25	VD2-31	VD2-32	VD2-33	VD2-34	VD4-11	VD4-12	VD4-13	VD4-21	VD4-22	VD4-24	VD4-31	VD4-32	VD4-33	VD4-35	VD2-11	VD2-13	VD2-14	VD2-21	VD2-23	VD4-23	VD4-25	VD4-34	VD4-36	VD4-02	VD4-03	VD2-22	VD2-01	VD4-01
																																			
**Type**																																			

A-VS1	0;1					2;3	0;1	0;1	1;2	1;2		0	1	1	0	2	1		3	0	2														
A-6BC	0					1	1	1	0	1		0	1	1	0	2	1		3	0	2														
A-8455	0					2	0	0	1	1		0	1	1	0	2	1		0	3															
B		0	1	0		1	1	1	0	1		1	0	1	1	1	0		3	0	2														
EB-E30		1	0		0	0	2				0	1	0	1	1	1	0			2	0														
EB-KKCP		0	1	1		2	2	1	0	1		1	0	1	1	1	0		3	0	2														
EB-859		1	0	1	3	1	1	1	0	1		1	0	1	1	1	0			2	0														
E		1	0		0	0	2				0	2	1	2	2	0	1	0		3	2														
C																								0;2							0				
D-NJ1																										0				0					
D-9N																										4									
F																									0				0			0			
R54																							0				0								
YP84																						0						0							
Mat116		2	2	2		2	0	0	1	1		0	1	1	0	2	1			1	1														
M56								1	1	0		0	1	1	0	2	1		3	0	2												0		
WC																																		0	0
1V					13																														
6N																										2									

### Optimization of microarray hybridization

To ensure the availability of sufficient amounts of target DNA for hybridization against a set of covalently bound oligonucleotide probes in a spatially accessible structure, amplification was conducted as duplex PCR. Using primer pairs VD1-f/VD2-r and 201CHOMP/ompA-rev, two biotinylated fragments containing VDs 1+2 and VDs 3+4, respectively, were produced. The resulting amplification products gave rise to hybridization signals at comparable intensity levels for VD2 and VD4 probes. In contrast, when PCR products comprising the entire *omp*A gene were hybridized, signals generated by binding to VD4 probes were significantly lower than those of VD2 probes (data not shown). This bias was compensated by doubling molar concentrations of the second primer pair in the above duplex PCR. Biotin labeling of target DNA via the use of 5'-biotinylated primers was preferred over internal labeling using biotin-dUTP because of higher sensitivity (data not shown) and lower cost.

To optimize the specificity of microarray hybridization, we systematically studied the influence of hybridization temperature (T_H_) and washing conditions. While hybridization patterns of the various genotypes were satisfactorily discernible at T_H _= 60°C, stringency was insufficient as indicated by poor resolution between signals of perfect-match probes and one-mismatch probes (data not shown). In contrast, the introduction of high-temperature (50°C) and low-salt buffer wash steps after both the hybridization reaction and incubation with the streptavidin-HRP conjugate led to high signal ratios of perfect-match vs. one-mismatch probes, so that single-nucleotide polymorphisms (SNPs) could be detected (see next paragraph). This high-stringency wash protocol even allowed T_H _to be lowered to 50°C in order to gain sensitivity.

### Microarray hybridization of type strains

**Figure 2:**
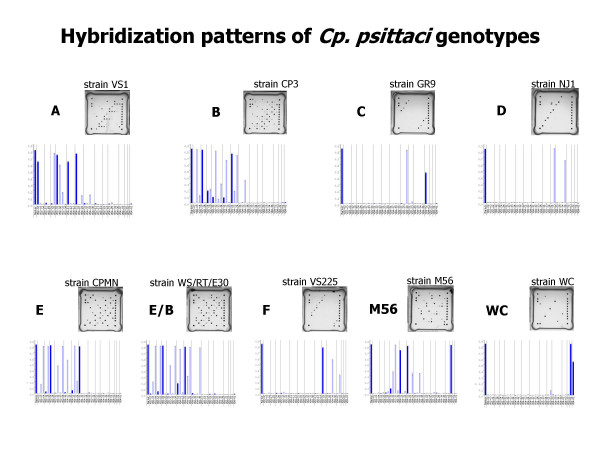
**Hybridization patterns presented as microarray images and barplot diagrams of nine strains representing *C. psittaci *genotypes A, B, C, D, E, E/B, and F, as well as strains M56 and WC. **The leftmost bar in each plot represents the signal of the internal staining control (biotinylated oligonucleotide probe).

**Figure 3:**
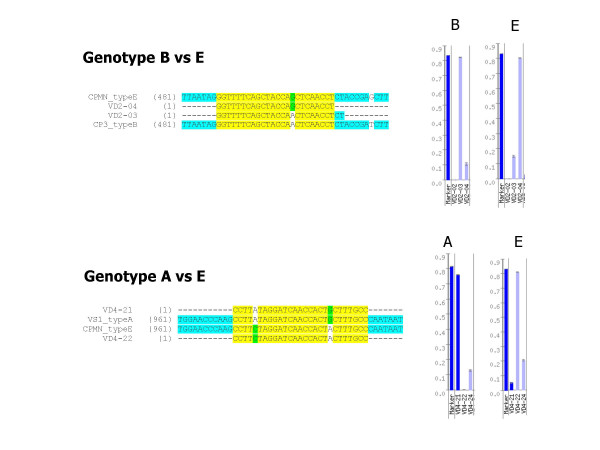
I**llustration of the specificity of the hybridization reaction on the AT microarray for genotyping of *C. psittaci*. **Alignments of target (*omp*A gene segments) and probe sequences are shown on the left-hand side, and the respective hybridization signals (including internal staining marker) are given on the right-hand side. Upper part: The signal generated by duplex formation at genotype E-specific probe VD2-04 is reduced to approx. 20% when the target has a single mismatch, such as genotype B. This applies also to genotype B-specific probe VD2-03, when reacting with genotype E. Lower part: Signal is reduced to less than 10% in the case of two mismatches on the target sequence.

Type strains of *C. psittaci *genotypes A, B, C, D, E, E/B, F, M56 and WC were examined using the AT *omp*A genotyping array. Barplots showing the distinct hybridization patterns are presented in Fig. 2. Comparison with theoretically expected, i.e. calculated patterns, revealed excellent agreement (data not shown) in all individual cases. Notably, regarding type strain patterns in the light of matching parameters given in Table 3, it could be confirmed that signal intensities of completely matching probes were significantly higher than those pertaining to probes having one or two mismatches to the target. For instance, Fig. 3 (upper part) illustrates that the signal of genotype B-specific probe VD2-03 with type B strain CP3 was more than 5 times stronger than with type E strain CPMN, which has a single mismatch in the target sequence. Conversely, the same applies to genotype E-specific probe VD2-04. Hybridization duplexes of targets and probes differing in two nucleotides were more than 10 times weaker than their perfect-match counterparts (see Fig. 3, lower part).

### Microarray hybridization of field isolates

A group of 12 field strains of *C. psittaci *was examined using the present AT microarray (Table 4). Genotypes were identified from the pattern of hybridization signals according to the matching scheme in Table 3. In addition, all strains were *omp*A sequenced, and genotypes were determined according to individual positions in the above global alignment. PCR-RFLP results are also given for comparison in Table 4. The findings of AT *omp*A genotyping are in complete agreement with the data of the other two tests.

**Table 4:** Genotyping of *C. psittaci *field strains using the AT microarray and comparison with PCR-RFLP and *omp*A sequencing

**Strain ID**	**Origin**	**Genotype identification by**		
		
		**PCR-RFLP**	***omp*A sequencing**	ArrayTube genotyping*
C1/97	sheep, Germany, 1997	C	C	C
C5/98	calf, Germany, 1998	A	A	A-6BC
C19/98	sheep, Germany, 1998	A	A	A-6BC
DC15	cattle, Germany, 2001	A	A	A-6BC
DC28	amazon parrot, Germany, 2001	A	A	A-VS1
DC29	amazon parrot, Germany, 2001	A	A	A-6BC
DC32	pigeon, Germany, 2001	B	B	B
Cal-10	ferret, USA, 1936	E	E	E
St04	chicken, Germany, 2005	A	A	A-VS1
St05	duck, Germany, 2005	E	E/B	EB-E30
St06	duck, Germany, 2005	A	A	A-VS1
St07	duck, Germany, 2005	E	E/B	EB-E30

* AT hybridization signal pattern assigned according to Table 3

## Discussion

The currently accepted system of genotyping for *C. psittaci *strains has evolved historically from its serological predecessor. The fact that serotypes have genotype equivalents [9], which has also been reported for, e.g., *E. coli *[14], proved to be helpful in the transition to the more accessible DNA-based typing methods, which are easier to standardize. However, given the serological history, it is not surprising that the *omp*A sequence analysis has revealed two notable limitations of the present genotype classification, i.e. the lack of complete coverage of naturally occurring strains and a general imbalance resulting from significant variations in genetic distances between individual genotypes. The latter reflects an inherent bias since the ideal panel would include genotypes genetically equidistant from each other.

Given the described deficiencies, the question about the usefulness of the present genotyping scheme inevitably arises. For instance, does the close genetic relatedness within the ABE cluster justify its subdivision into the four genotypes A, B, E and E/B?

The authors are of the opinion that there are important arguments in favor of maintaining the present classification and nomenclature, provided it is amended by a few adjustments and extensions.

i) Despite the amazing genetic heterogeneity displayed by *omp*A sequence database entries, it seems that the vast majority of field strains belong to the ABE cluster. This is indicated by several published studies [18,10,11,19], by the data presented in Table 4 and also the long-term experience of the authors' laboratories (data not shown). The small group of so far untyped isolates appears to represent only a small proportion of naturally occurring strains.

ii) Each genotype should be defined by a representative reference strain and its complete *omp*A sequence. While a genotyping system based on a single gene may not appear sufficiently comprehensive in the age of genomics, it should be noted that *omp*A encodes the major protein antigen of chlamydiae, and even the minor sequence variations including SNPs are mostly translated into different amino acids (and, potentially, different epitopes). The fact that genotype-specific antigens can be distinguished by specific MAbs indicates that these differences do matter in the context of immunogenicity, host preference, virulence and, thus, epidemiological importance. This is why we suggest maintaining the currently accepted genotypes, including the closely related ones of the ABE cluster.

iii) To account for the natural variability of *omp*A sequences in *C. psittaci *strains, emerging branches of untyped isolates should become provisional genotypes until their epidemiological relevance and representative status are proven. To account for intra-genotype variability, we suggest that heterogeneous genotypes, such as A, E/B and D, should be further divided into subgroups named after a typical representative, e.g. A-VS1, A-6BC, etc. (see Table 1). This approach will ensure openness and flexibility of the genotyping scheme.

iv) The amended genotyping scheme should be re-evaluated and overhauled when a sufficiently large number of complete genome sequences of *C. psittaci *strains becomes available.

If *omp*A genotyping of *C. psittaci *is to be conducted in the framework of surveillance and monitoring studies, the performance of PCR-RFLP will be insufficient because of its limited sensitivity and inability to characterize atypical strains. As mentioned above, serological typing is limited to only six serotypes, but is not amenable to high-throughput examination and it lacks a strictly molecular basis. The real-time PCR procedure proposed by Geens et al (2005) can be very sensitive and specific for identification of seven of the established genotypes, but it is rather laborious and expensive because each sample has to be examined in seven individual runs. Sequencing of the complete *omp*A gene should serve as the gold standard for genotyping of *C. psittaci *since the respective DNA sequence contains all details necessary for genotype identification and the data can be stored in public databases to be easily accessible to researchers and diagnosticians. However, due to cost and labor intensiveness, it is not feasible at present to use DNA sequencing of this 1212-bp gene as a routine procedure in all diagnostic laboratories.

Being more rapid and economical, the AT microarray assay developed in the present study represents a powerful tool for sequence-based, sensitive and reproducible high-throughput genotyping. In principle, the procedure consists in parallel probing of 35 different targets and, in view of the recognition of minor nucleotide sequence variations including SNPs, amounts to re-sequencing the discriminatory regions in VDs 2 and 4 of the *omp*A gene. Future use of the AT *omp*A genotyping assay will enable diagnosticians in human and veterinary medicine to trace epidemiological chains, explore the dissemination of the various genotypes and other strains of *C. psittaci*, as well as identify new representatives of this amazing pathogen.

## Conclusion

According to the data of *omp*A analysis, *C. psittaci *is genetically the most heterogeneous species of the genus *Chlamydophila*. The traditional genotyping system should be amended and extended because it does not adequately reflect the extent of intra-species heterogeneity. Serology, PCR-RFLP and real-time PCR are unable to discriminate among all currently accepted genotypes and identify strains of new types. While genotyping based on complete *omp*A sequences should serve as gold standard, many of the smaller laboratories may not be able to use it in routine diagnosis. The results of the present study have demonstrated that the newly developed DNA microarray-based *omp*A genotyping assay represents a promising diagnostic tool.

## Methods

### Chlamydial strains

Genomic DNA of the following reference strains, each representing a particular genotype, was used to generate master patterns for the *omp*A genotyping assay:

VS1 (genotype A, from parrot, USA, 1985), CP3 (genotype B, from pigeon, USA, 1957), GR9 (genotype C, duck, Europe, year unknown), NJ1 (genotype D, from turkey, USA, 1959), CPMN (genotype E, ferret/human, USA, 1934), VS225 (genotype F, parakeet, USA, 1991), all of which were described in reference 5 and obtained from the chlamydia strain collection of AFSSA, Maisons-Alfort, France. Strains WS/RT/E30 (genotype E/B; isolated from a duck, Germany, 2001, reference 10), WC (from cattle, USA, 1990, reference 7) and M56 (from muskrat, USA, 1961, reference 7) were kindly provided by Daisy Vanrompay, University of Ghent, Belgium.

In addition, the field isolates given in Table 4 were examined in this study.

### Sequencing of the *ompA *gene

The complete gene was amplified by PCR using primers CTU (5'-ATG AAA AAA CTC TTG AAA TCG G-3') and ompA-rev (5'-TCC TTA GAA TCT GAA TTG AGC-3') using the following cycling profile: initial denaturation 96°C for 60 sec, 40 cycles of 96°C/15 sec, 50°C/60 sec, 72°C/60 sec, final extension 72°C for 60 sec.

Products were electrophoresed in 1% agarose gels, and the specific bands of approximately 1200 bp were excised with a scalpel and DNA extracted using the innuPREP Gel Extraction Kit (Analytik Jena, Jena, Germany). DNA sequencing was carried out by cycle sequencing using the BigDye™ Terminator Cycle Sequencing Ready Reaction Kit (Applied Biosystems, Darmstadt, Germany) according to the instructions of the manufacturer. The following primers were used: CTU, VD1-f (5'-ACT ACG GAG ATT ATG TTT TCG ATC GTG T-3'), VD2-r (5'-CGT GCA CCY ACG CTC CAA GA-3'), CHLAGEN-1 (5'-CGG CTG CAT TCA ACT TGG-3'), 201CHOMP (5'-GGI GCW GMI TTC CAA TAY GCI CAR TC-3'), and ompA-rev. Nucleotide sequences were determined on an ABI Prism 310 Genetic Analyzer (Applied Biosystems).

### *In silico *analysis of *ompA *sequences

All available sequences of the *omp*A gene of *C. psittaci *were downloaded for analysis from the database of the National Center for Biotechnology Information (NCBI). A total of 210 sequence entries were found (by the date of manuscript submission). Of these, 89 sequences shorter than 500 nt were excluded from further analysis for failing to cover all four variable domains. Of the remaining 121 entries, 68 were found to belong to *C. psittaci *and 53 to other *Chlamydophila *species. Only 25 sequences from *C. psittaci *and 28 from other *Chlamydophila *spp. were found to include the full coding sequence (CDS) of approximately 1212 nt. In contrast, 70 entries lacked terminal parts of the CDS, but were retained because for some genotypes not a single complete sequence was available. All items were included in a global *omp*A sequence alignment using the program E-INS-I of the MAFFT package [20].

Classification was done first by visual inspection of the alignment using Clustal X [21] and subsequently by calculating a sequence similarity matrix (see Additional file 1), from which a split network graph was constructed (Fig. 1) using the program SplitsTree4 [22].

Before starting the similarity matrix calculation, redundant items were removed, i.e. any sequence that could be retrieved under another sequence was deleted, and only unique sequences were kept. The sequences were brought to identical length by cropping highly conserved 3' ends in order to avoid distorted results due to alignment of differently sized sequences. Thus, the analyzed segment comprised 992 nucleotide positions (median sequence length 942 nt) including all four variable domains. The final set contained 63 unique sequences.

### Microarray design

The present array carries 35 oligonucleotide probes recognizing targets from VD2 and VD4 of the *omp*A gene of *Cp. psittaci*. Nucleotide sequences of all probes are provided in Table 2. The oligonucleotides had the following features: average size 26 nt (22 – 30), melting temperature 60.3°C (59.7 – 61.2), G+C contents 46.0 mol-% (37.0 – 59.0). Each probe sequence was subjected to local BLAST analysis against all known *C. psittaci omp*A sequences to verify the respective genomic target and rule out unwanted cross-reactions. Biotinylated oligonucleotide probes were added to monitor the staining reaction, mark the corners of the array and facilitate normalization of signal intensities. Each genotype probe was spotted four-fold, the controls 15-fold, thus bringing the total number of spots on the array to 155. Fabrication of the AT microarrays was described previously [12].

### DNA extraction

Cell cultured strains were DNA extracted using the High Pure PCR Template Preparation Kit (Roche Diagnostics, Mannheim, Germany).

### Biotinylation PCR and AT hybridization

Target DNA was amplified and biotinylated by duplex PCR using two pairs of *omp*A primers covering all variable domains. Primer pair VD1-f and VD2-r (sequence as above, but 5'-biotinylated) gives rise to a 418-bp product which includes VD1 and VD2, whereas primers 201CHOMP and ompA-rev (sequence as above, but 5'-biotinylated) defined a product of 570 bp covering VD3 and VD4. The temperature-time profile was: initial denaturation at 96°C for 60 sec, 40 cycles of 94°C for 30 sec, 50°C for 60 ssec, 72°C for 30 sec, and final elongation at 72°C for 240 sec.

Each 20-μl reaction contained 10 pmol of the first and 20 pmol of the second primer pair, as well as 1 mM each of dNTP mix, 1 U Taq DNA polymerase (Fermentas, St. Leon-Roth, Germany), 2 μl 10 × PCR buffer (Fermentas), 1 μl 50 mM MgCl_2_, and 1 μl of the DNA template.

The AT vessel was conditioned by washing with 500 μl each of deionized water and Hybridization buffer 1 (Clondiag Chip Technologies, Jena, Germany) at 50°C for 5 min. All incubations were carried out upon slight shaking (550 rpm) on a heatable horizontal tube shaker (Thermomixer comfort, Eppendorf, Cologne, Germany). For denaturation, 1 μl of the 5'-biotinylated PCR product was diluted with 99 μl hybridization buffer in a separate tube, heated at 95°C for 5 min and put on ice. After transfer into the AT, hybridization was allowed to proceed at 50°C for 60 min. The supernatant was then discarded, and the array was washed consecutively with 500 μl 2 × SSC/0.01% Triton X-100 (50°C, 5 min), 500 μl 2 × SSC (50°C, 5 min), 500 μl 0.2 × SSC (50°C, 5 min), and 500 μl 0.2 × SSC (35°C, 5 min). Vacant binding sites of the microarray were blocked by incubation with a 2% solution of Blocking Reagent (Roche) in hybridization buffer at 30°C for 15 min. Subsequently, the AT was incubated with 100 μl of a 1:5000 dilution (0.5 μg/ml) of streptavidin-peroxidase polymer (Sigma-Aldrich, Taufkirchen, Germany) at 30°C for 15 min followed by three wash steps, i.e. 500 μl 2 × SSC/0.01% Triton X-100 (30°C, 5 min), 500 μl 2 × SSC (20°C, 5 min), 500 μl 0.2 × SSC (20°C, 5 min). Finally, 100 μl of the peroxidase substrate Seramun Grün (Seramun Diagnostica, Heidesee, Germany) were added. Hybridization signals were measured using an ATR-01 transmission reader (Clondiag) and processed using the Iconoclust software, version 2.3 (Clondiag).

Normalized intensities of the spots were calculated according to the following equation:

NI = 1 - (M/BG), with NI being the normalized intensity, M the average intensity of the spot, and BG the intensity of the local background. Values range from 0 (no signal) to 1 (strong signal).

### PCR-RFLP genotyping

Extracted DNA was amplified by PCR using primers CTU/CTL, and the product was subjected to restriction enzyme digestion using *Alu*I and *Mbo*II as described by Sayada et al. [8]. Following agarose gel electrophoresis, cleavage patterns were compared with those of the reference strains for genotypes A, B, C, D, E and F.

## Availability and requirements

National Center for Biotechnology Information (NCBI): 

## Authors' contributions

KS conceived of the study and coordinated it, participated in bioinformatic work, supervised laboratory work and drafted the manuscript. KL conducted PCR-RFLP testing and provided most of the reference strains and their DNA. HH conducted *omp*A sequencing and participated in the design of the study. ES conducted cell culture of the chlamydial strains used. RE participated in the design of the study, as well as in the sequence alignment and further analysis, and supervised part of the laboratory work. PS participated in designing the study and in bioinformatic work. All authors read and approved the final manuscript.

## Supplementary Material

Additional file 1 Sequence similarity matrix. Calculated similarity matrix of all unique *omp*A sequences of *C. psittaci *strains, on which the split network graph in Fig. 1 is based. Click here for file

Additional file 3 Basic data of *C. psittaci *strains included in Fig. 1 and the final *omp*A sequence alignment. Basic data of *C. psittaci *strains and GenBank accession numbers of *omp*A sequences included in the graph in Fig. 1 and the global *omp*A sequence alignment in Additional file 2 Click here for file

Additional file 2 Global alignment of *omp*A sequences found in the GenBank database. Alignment of 63 unique *omp*A sequences of *C. psittaci *strains from the GenBank database (992 positions) Click here for file
